# On the Goals and Limitations of Psychological Science: Some Thoughts in Memory of Daniel Kahneman

**DOI:** 10.1177/17456916261417988

**Published:** 2026-07-06

**Authors:** Gideon Keren

**Affiliations:** Department of Social Psychology, Tilburg University

**Keywords:** psychological science, goals, methodology, models, narratives, Daniel Kahneman

## Abstract

Daniel Kahneman was a prominent multifaceted psychologist whose work had a persuasive impact on the fields of attention (his initial research) and the field of judgment and decision-making (and the closely related domain of behavioral economics), for which he received the Nobel Prize. The current article was initiated by a correspondence with Kahneman regarding the scientific value of the two-system premise. This correspondence went much beyond the initial two-system issue, ending with a query regarding the methods and goals of psychological research and its inherent limitations. The major issue concerned the extent to which precision in psychological research can be achieved and, specifically, the value of formal models in psychological science. This article summarizes some fundamental controversial issues raised in this correspondence regarding the nature of knowledge attained in psychological science and the role of theories and models in the process of obtaining this knowledge.

“We have different views of what science is about. You think progress is formulating precise theories. I think progress is making and describing interesting discoveries” (D. Kahneman, personal communication). This is the first sentence of a long correspondence I had with the late Daniel Kahneman, one of the more prominent psychologists in the past half century, following the publication of my critical comments regarding “two-system” theories (e.g., Keren, 2013; Keren & Schul, 2009). Although our correspondence was triggered by the controversial two-system issue (for a summary, see Melnikoff & Bargh, 2018), it ended with an analysis of broader questions regarding what are sensible and realistic goals to be achieved by psychological science and by which means. The current article summarizes the main points of this correspondence and briefly assesses some possible guidelines for future developments of the field.

## Precision Impossibility

I begin with Kahneman’s comment questioning whether precise theories are “the name of the game.” In the current article, precision is construed as the coarseness underlying psychological research, specifically as related to measurement (how close measurements of the same identical item or construct are to each other). In my first reply, I started by noting that a long time ago I realized that the strict scientific requirements that may be appropriate in the exact sciences, specifically in physics, are not fully applicable (probably impossible) to psychology. Thus, broadly speaking, I agreed with Kahneman, who in his first letter of our correspondence claimed that attempts to obtain precision are fruitless because precision is not possible in psychology.

There are several reasons why literal (comprehensive) precision is unattainable in psychology. One account for this impossibility (or perhaps a reflection of it) is based on the large amount of noise associated with human behavior, as convincingly demonstrated by Kahneman in his last book, *Noise* (Kahneman et al., 2021). Although the book focuses mainly on noise in human judgments (e.g., physicians’ diagnoses, decisions of judges in court, assessments of job candidates, fingerprint analyses), it is unquestionably generalizable to almost any human behavior. Even in highly controlled experiments in psychology, there is always variability^1^ that researchers aim to control by trying to keep all variables that are not under investigation fixed (or using large samples for minimizing variability). However, strict controls overlook the potential influence of other variables and various interactions. Hence, when applied to behavior outside the laboratory, noise increases dramatically, as demonstrated by Kahneman and his colleagues.

Variability implies that generalizations from experimental results to real-world phenomena are not straightforward (and are at best probabilistic). Further, experimental data need to be statistically analyzed and interpreted, yet, as noted by Cohen (1990), “there is no royal road to statistical induction,” “the informed judgment of the investigator is the crucial element in the interpretation of data,” and “things take time” (p. 1304). In short, each researcher’s informed judgment may govern the setting of the relevant parameters, and, similarly, informed judgment determines the researcher’s interpretation and conclusion.

## Factors Hampering Precision: Conceptual Vagueness and Measurement Difficulties

Two major related accounts for the lack of precision in psychology concern the lack of unequivocal definitions of psychological constructs and the difficulties associated with their measurement. Psychological constructs are regularly illusive—imprecise and not well-defined (e.g., Danziger, 1997). Keren and Breugelmans (2020) claimed that psychologists use psychological constructs as if they were precise and well-defined, although this is generally not the case. There is evidently a gap between poorly defined abstract concepts (e.g., “utility,” “happiness,” “risk,” “regret”), their psychological operationalization, and their experimental definition. The deficiency of an exact definition of most psychological concepts leads to major measurement difficulties and consequently to the lack of envisioned precision. Even Allan Turing, one of the initial computer inventors, was aware of the elusiveness of concepts (Turing, 1950). Asking whether “Can machines think?” he noticed that the terms “machine” and “think” could not be accurately defined and thus that the question in some respects was meaningless. (Instead, he suggested a closely related question that he proposed to answer by a game termed “the imitation game,” which supposedly may provide some reasonable insight.)

Hutmacher and Franz (2025) correctly noted that “psychological concepts are inherently vague in the sense that their meanings and the rules for their application are indeterminate” (p. 2). This does not mean that psychological constructs lack sense—in daily communication we all understand the meaning of terms such as “rationality,” “utility,” “regret,” “attention,” and so on; however, accurate definitions of most concepts are inaccessible and hence not measurable. For instance, “IQ” is not a well-defined concept unless one states that “IQ is what intelligence tests measure,” which is obviously a circular definition. In daily communication, however, it is a way to express a general mental and intellectual capability.

Another example from the fields of behavioral economics and decision-making concerns the term “utility.” “Utility” is a nonconcrete term, and although we use this term in our communication we do so as if its meaning is supposedly known and understood in the same way by everyone. Essentially, it can never be defined in exact and unambiguous terms. Kahneman, who in collaboration with Amos Tversky (Kahneman & Tversky, 1979; Tversky & Kahneman, 1992) developed prospect theory as a possible substitute for expected utility theory, became aware at a later stage in his life of the drawbacks and limitations of both theories, about which I elaborate on later.

As a final example, consider the concept of “subjective well-being” (e.g., Kahneman & Krueger, 2006), which is multidimensional and thus can be grasped in different ways depending on which dimensions are accentuated. Many researchers implicitly assume that the concept is equally grasped by everyone and can supposedly be precisely assessed; however, this is highly questionable. On the basis of several large-scale studies, Wojcik et al. (2015) concluded that these studies “illuminate the contradictory ways that happiness differences can manifest across behavior and self-reports” (p. 1243).

Because of the equivocal nature of psychological concepts, theories are open to more than one interpretation and can be tested in multiple ways. One crucial reason for this state of circumstances is embedded in Robyn Dawes’s observation that, unlike in physics, there is no psychological reality, yet, evidently, psychologists often tacitly assume its existence (Dawes, 1972). One may argue (as did a reviewer of this article) that psychological concepts may represent an individual’s internal reality, which exists in the mind of the beholder. However, researchers have no access to an individual’s mind, not to mention that different individuals may have diverse interpretations of the same state of affairs or same concepts, leading to somewhat different perceived realities. Consequently, there is no single definition of most psychological concepts, and precision, as claimed by Kahneman, is impossible.

Physics directly relates to a (shared) reality; measurements such as heat (Celsius, Fahrenheit, or any other scale) or distance (in miles or kilometers) are unidimensional,^2^ well-defined, and thus meaningful and easy to comprehend. Psychological concepts, in contrast, do not relate to an existing (shared) reality and are typically multidimensional, and the meaning of their measurement is questionable. This is probably one of the main reasons why psychological concepts are characterized by variability and noise (Kahneman et al., 2021), whereas physical measurements—at least Newtonian physics—are plain and considered unequivocal (but for a different perspective, see Michell, 2005). Indeed, as Trafimow and Fiedler (2024) noted, “There is a trepidation, anxiety, or intuition, which has persisted for more than a century, that psychology theories are less anchored in fundamental laws than physics theories” (p. 439).

A consequence of the frail precision of psychological concepts concerns the inability to measure, in a reliable and authentic way, any psychological construct beyond an ordinal scale (e.g., Franz, 2022). Ordinal scales contain limited information, yet most researchers analyze and evaluate results as if they were on an interval or even ratio scale (on the use of “dominance” statistics to answer ordinal questions, see Cliff, 1993). Much creativity and productive work on measurement theory was conducted in the 1970s and 1980s and was summarized in a series of three volumes (Krantz et al., 1971; Luce et al., 1990; Suppes et al., 1989). However, as noted by Cliff (1992) and Falmagne (1992), this work had little influence on the development of scientific psychology. In the past couple of decades, measurement issues in psychology have been largely overlooked by researchers, and most psychological research was conducted as if there were no measurement problem, namely falsely assuming and using an interval or ratio scale. A possible reason for Kahneman’s disappointment with and criticisim of prospect theory was the realization that the implicit measurement assumptions of the theory are unrealistic.

## Models, Theories, and Paradigms

Kahneman was acutely aware of the measurement limitations and their consequences, which was supposedly a main reason for his doubts concerning precise theories and models, specifically in psychology. In an unfinished, unpublished manuscript, he critically evaluated prospect theory, which he had developed 40 years earlier with Tversky (Kahneman & Tversky, 1979). His manuscript demonstrated a thoughtful approach to science, not hesitating to critically evaluate his own scientific work.

A major question raised in this unfinished manuscript was what makes a theory accepted and respected. Answering this intricate question is beyond the scope of the current article. However, some major desired features of an appropriate theory or model may be noted, namely that it (a) highlights the essential variables underlying the investigated phenomenon while suppressing the minor or irrelevant ones; (b) is accurately expressed (ideally with a mathematical model); and (c) attempts to elucidate—implicitly or explicitly in terms of cause-effect relations (Hogarth, 1986)—an interesting notion or observation.

Examining prospect theory, Kahneman claimed that a major contribution of the theory was to highlight the concept of “loss aversion,” which states that gains and loss perspectives are assessed asymmetrically (e.g., the pain of losing $1,000 is comparatively larger than the joy of gaining $1,000). However, he further noted that “loss aversion can be stated in a few words without the mathematical machinery of prospect theory, and it could surely have been presented in a different form, perhaps in an essay” (indeed, loss aversion had been presented long before the appearance of prospect theory; Peeters, 1971). Kahneman concluded that “the community of scholars would have ignored loss aversion if it had been presented as an isolated proposal or as part of a discredited theory.”

Kahneman’s doubts regarding formal models were not restricted to prospect theory. In another unpublished manuscript, “Notes on an Expanded Paradigm,” he attempted to assess the overall scheme of decision-making research, noting that “human behavior is too rich and complex to be understood without first oversimplifying it. How to simplify is the question.” He proposed that a simplifying approach is adopting “a particular setting as a model for a much broader class of human situations, and to study behavior in that setting as a model of action in the broader domain.”

For example, behavioral decision-making is dominated by the so-called gambling paradigm, according to which studying choice among gambles is considered a proxy for studying choice behavior in general. The decision maker is assumed to be “a bounded rational gambler engaged in selecting the most advantageous bet from a small set currently available.” He further noted that a general paradigm^3^ makes it easy to perceive and represent certain real-world phenomena yet may also make it harder to represent other aspects of reality that are suppressed. For instance, the gambling paradigm centers on the cognitive reasoning processes associated with the choice between tangible outcome alternatives described with well-known specified probabilities (by itself a questionable assumption); in contrast, it suppresses potential intangible consequences such as emotional and anticipatory emotional aspects (e.g., fear, hope, regret) and entirely ignores intangible outcomes. As another instance, the gambling paradigm strictly highlights maximization, whereas in real life attempting to satisfy may often be more realistic.

Although he noted that the gambling paradigm enables the development of precise mathematical models, Kahneman questioned its validity. He explicitly stated that he and Tversky “thought of ourselves as studying choices of gambles as a proxy for studying other decisions. Many years later, I am less sure of what we were doing.” He thus questioned external validity of the paradigm, which serves as a main foundation of expected utility theory, a point that was often overlooked (see Lopes, 1981) or discounted too frequently.

The supposition that people view their decisions as gambles is unequivocally unrealistic, as is the assumption that people know and calculate probabilities. For instance, Von Neumann and Morgenstern (1944), who laid the modern basis for expected utility theory, noted that

probability has often been visualized as a subjective concept more or less in the nature of an estimation. Since we propose to use it in constructing an individual, numerical estimation of utility, the above view of probability would not serve our purpose. The simplest procedure is, therefore, to insist upon the alternative, perfectly well-founded interpretation of probability as frequency in the long runs. This gives exactly the necessary numerical foothold. (p. 10)

To their credit, Von Neuman and Morgenstern explicitly stated their improbable assumption. Like Von Neuman and Morgenstern, Savage (1954) distinguished between the actual real world with all its complexities (utterly impossible to envisage) and the small, simplified (imaginary) world underlying models and theories.

Although Kahneman did not refer to Von Neuman and Morgenstern’s robust (but probably unrealistic) assumption, I suppose that such examples led him to doubt the exact nature of the contribution of mathematical theories in psychology. In his unpublished manuscript (Kahneman), Kahneman mentioned meeting the editor of *Econometrica* who had accepted the prospect theory article, in line with my speculation:

I asked him what had led him to accept a paper by two psychologists. I was hoping he would cite one of the ideas, perhaps loss aversion which is my personal favorite, but he smiled as he answered “I liked the math.” He was only half-joking.

Parenthetically, Kahneman’s reservations regarding the overvalue of strict and precise mathematical models is not restricted to psychology. In a thought-provoking book entitled *Lost in Math: How Beauty Leads Physicists Astray*, the physicist Sabina Hossenfelder claimed that theoretical physics focuses too much on the elegance and beauty of mathematical models while failing to comprehend the underlying physical phenomenon on a deeper level (Hossenfelder, 2018). Put differently, the laws of nature are supposedly more complex and do not necessarily follow the elegance, simplicity, and beauty of mathematical models. Kahneman came to a similar conclusion regarding the field of judgment and decision-making, claiming that “I no longer believe that it is possible to formulate a theory of even moderately complex gambles that is precise, true and tolerably simple.”

## Theories and Models Versus Interesting Observations and Stories

Returning to the lack of perfect precision, my correspondence with Kahneman raised the question regarding the pros and cons of two overall approaches of studying psychological phenomena. One scheme is based on the abstraction of observations and correspondingly developing a theory or a model, preferably a mathematical one. A model is invented on the basis of (explicit) strict assumptions, formally resulting in more exactness. Yet, concurrently, the generalizations that can be derived from the model are confined. In addition, any model contains a translation problem, namely how to go from theoretical concepts to real-world concepts. Advocates of using theories and models accentuate their benefit, namely that they enable an accurate formulation of hypotheses subsequently tested by comparing derived predictions against obtained data (usually by statistical tools). Yet the coherent testing procedure of models does not solve the complicated process of conversion to real-life settings.

Although I agreed with Kahneman that perfect precision, certainly in psychology, is impossible, it clearly does not imply that theories and models are fruitless. On this last point, our opinions ultimately somewhat departed. Theories and models provide an outline for revealing hypotheses and rigorously testing them. Hogarth (1986), for instance, considered models to be working hypotheses expressed in causal-effect terms, which are in his opinion useful because “(a) economy of description, and (b) the power to suggest implications that are not evident at first sight” (p. 443). Most likely, Kahneman was not unaware of the potential benefits of models but was probably concerned that they were accessible only to a small number of individuals.

The alternative scheme, as proposed by Kahneman, is to describe what he referred to as “interesting discoveries” by a compelling story. Interesting discoveries can supposedly be obtained by incidental or intensive—not necessarily strictly controlled—observations. But is there a universal and unambiguous definition of what constitutes a compelling story? To evade misinterpretation, I quote Kahneman at length.

Psychological stories, in my terms, are ways of putting things together in chunks that work well in people’s mind, terms that come to mind at the right time and help make sense of events – usually by highlighting commonalities that are otherwise missed. I think much useful psychology is done in that vein, and the measure of success is the number of new observations that are predicted, and the satisfaction of understanding (yes, that may sound a bit like literature). I have spent or mis-spent my life telling this kind of stories and proposing this kind of concepts. Mental effort, representativeness, loss aversion, attribute substitution, the psychology of counterfactuals. . . . None of these is satisfactory Science – but the evidence suggests that they have been useful to many people, even if others despise them. Perhaps the best example is the reference point of prospect theory, which was left largely unspecified. Many people with a taste for precision have objected to it, but I think it has been a very useful notion. (D. Kahneman, personal communication)

“Interesting discoveries,” using Kahneman’s term, can be explored in more than one way. A common mode is the use of a well-controlled experimental design to test a specific account or elucidation of an interesting discovery, such as an empirical test of loss aversion and the potential underlying variables that affect it (e.g., Schmidt & Traub, 2002). An alternative approach is by demonstrating the existence of an interesting discovery. Sugden (2005) referred to demonstrations as an exhibit that contains “an experimental design which reliably induces a surprising regularity, typically combined with an informal hypothesis about the underlying causal mechanism” (p. 291). For instance, Asch’s (1956) conformity experiments provide a clearcut demonstration of conformity. Regardless of the possible methodological problems and the exact interpretation of the results (e.g., Friend et al., 1990), the experiments demonstrate, unequivocally, the existence of conformity. Similarly, notwithstanding ethical and methodological criticisms, Milgram’s (1963) study reveals the obedience phenomenon. Note that demonstrations are not explanations; both Asch’s and Milgram’s experiments should be viewed as demonstrations and not explicit accounts.

Asch’s and Milgram’s experiments are distinctive in that they demonstrate how the respective phenomena can be obtained, by surprisingly easy means and under easy circumstances, that are, perhaps naively, believed to be immune to conformity and obedience. The experimental settings supposedly illuminate an essential psychological concept (“interesting discoveries” in Kahneman’s terms) and can be further used by researchers for exploring each phenomenon’s limits and potential means of moderating it. In other words, Kahneman’s point is that interesting observations and demonstrations are a good starting point, after which researchers may follow with more systematic and controlled experiments.

Even the original pioneering research of Tversky and Kahneman (Kahneman et al., 1982; Tversky and Kahneman, 1974) that introduced judgmental biases such as representativeness, availability, and adjustment and anchoring may be construed as exhibits rather than explanations of the biases. The biases were interpreted by Tversky and Kahneman as errors resulting from cognitive shortages, whereas others wondered whether the heuristics should indeed be viewed as failures of rationality. For instance, Gigerenzer (1991, 1996) questioned whether the assumptions of rational theory are always met (in reality) and proposed that the heuristics may often be viewed as adaptive and useful tools. Tversky and Kahneman’s original demonstrations of the heuristics are undoubtedly useful observations and interesting discoveries. Yet a literature review suggests that their interpretation and underlying processes remain equivocal (e.g., Keren & Teigen, 2004).

Inspiring observations can be outlaid not just by exhibits but also by a narrative. Kahneman thought that narratives enabled lumping things together, leading to a clear comprehension in people’s mind. As examples he mentioned the idea of mental effort (Kahneman, 1973; but see reservations by Bruya & Tang, 2018), or the reference point of prospect theory. According to prospect theory, the reference point determines how an outcome is perceived and evaluated, yet no theory on the exact location of the reference point exists. Kahneman noted that although the concept is largely unspecified, it is nonetheless a useful one.

Unlike experimental exhibits, stories attempt (explicitly or implicitly) to offer explanations. However, a narrative can be framed in different ways and can be grasped in more than one way (thus leading to different inferences and predictions). Moreover, a story can be misinterpreted and may even result in unwarranted understandings. For instance, Epstein (2018) examined psychologists’ narrative that, according to him, sloppily claims the analogy between the human’s brain and the computer. Whereas computers undeniably process, store, and retrieve information and have real physical memories, the brain, he noted, “does *not* contain most of the things people think it does – not even simple things such as ‘memories’” (para. 1). Epstein further noted that information processing is “a metaphor, after all, just another metaphor – a story we tell to make sense of something we don’t actually understand” (para. 20).

In a similar vein, Kahneman (2011), using the two-system narrative (referring to them as two characters), warned the reader about the two-system fictitious nature: “System 1 and system 2 are not systems in the standard sense of entities with interacting aspects or parts. And there is no one part of the brain that either of the systems would call home” (p. 29). Unfortunately, many researchers using the two-system vocabulary have ignored its fictitious nature (e.g., Keren & Schul, 2009; Melnikoff & Bargh, 2018).

Kahneman thought that the two-system terminology was a useful fiction and claimed that the two-character story (i.e., Systems 1 and 2) provided readers “with a way to make sense of patterns in their behavior and in the behavior of others. Do you think that the (openly admitted) fact that this framework is a story rather than a cleanly testable theory makes it completely useless?” Kahneman, in my opinion, was certainly right in suggesting that a story’s context may offer a useful framework for facilitating comprehension, yet he probably underrated the possible perils of wrongly interpreting a story (in different ways). For instance, a likely misinterpretation concerns taking inessential story elements literally, such as assuming that the two systems are real.

The two schemes (theories and models or stories) are in some respects incompatible yet contain some commonalities (and are occasionally complementary). Specifically, both supposedly offer an outline in which phenomena are perceived as coherent and understood in causal terms. The two schemes contrast considerably in how they try to construct an understandable framework and the confines they impose on the proposed framework. In other words, they differ in how the boundaries on the presumed framework are specified and imposed. Stories contain much fewer restrictions; these are implied from the story’s content and structure and are concealed rather than explicitly stated. A story’s minimal constraints, almost by definition, makes it less precise and accurate regarding the phenomenon it attempts to analyze. Diverse individuals may emphasize different aspects of a story, potentially comprehending it in different ways.

The two schemes also differ in the means by which their elucidation of the phenomena under investigation are tested to be authentic and true. Although theoretical models are strictly designed and evaluated by comparison with data specifically designed for that comparison, stories are not inherently constructed for comparisons (because there are several alternative stories) but are rather assessed by their “goodness,” namely the extent to which they portray a sound and convincing causal narrative. Although model-data evaluation is mainly done by well-defined statistical analyses, the “goodness” of a story is an equivocal term that is subjective and not well-defined. A concrete story is appealing because it is easier to envision the context(s) in which the phenomenon may occur and facilitates comprehension. However, a story is constructed by words and is thus susceptible to alternative interpretations, as demonstrated in numerous studies of framing (e.g., Keren, 2011; Tversky & Kahneman, 1981). Unlike theories or models, there is no strict method for testing the accuracy or veracity of alternative interpretations. There is an implicit (sometimes explicit) trade-off between accuracy and generalizability that is highly correlated with a number of restrictions. The major drawback of stories is that they are more (sometimes too) flexible: They lack commitment to a general definition of concepts (e.g., the interpretation of “risk” may differ in different stories and may be differently comprehended by different people); even a coherent story may miss some indispensable factors (variables) that are supposedly essential for suitably portraying the phenomenon of interest.

The two schemes are somewhat related to Bruner’s (1984) distinction between two modes of thought, which he termed the “paradigmatic” and the “narrative” (Bruner’s distinction between “intuition” and “reasoning” should not be confused with the two-system conceptual framework). The paradigmatic (or logico-scientific) mode is based on consistency and noncontradiction; it establishes truth “by appeal to formal verifications procedures and empirical proof” (p. 1). The narrative mode, in contrast, establishes what Bruner termed “truth-likeness,” or verisimilitude. Although the cornerstone of the paradigmatic mode is the possibility of falsifiability, in line with Popper (1968), the hallmark of the narrative mode is believability or informal sensemaking. Bruner further maintained that each mode offers a partial understanding of the psychological world from a different vantage by using different methods that supposedly tap into different facets of our comprehension. Conceivably, a similar conclusion may be drawn regarding the knowledge provided by models (theories) and stories.

What, then, are the alternatives given that almost any psychological phenomenon is complex and affected by many variables? Models, including mathematical ones, allegedly simplify by stating the proper limitations and focusing on the two or three most prominent variables underlying the phenomenon under investigation. Yet Dawes (1999) correctly claimed that a model also requires “a good story appended to it” (p. 29). Specifically, he noted the difficulties associated with relying on probabilistic contingencies, or comprehending a model, unless they are embedded in a convincing causal story. For instance, in the case of the expected utility (EU) model, he noted that

people cannot conform (“descriptively”) to the standard EU model, given that probabilities often cannot be combined either implicitly or explicitly in the absence of a good, clearly relevant story justifying the combination. Moreover, that inability severely limits the standard EU model for use in prescriptive decision making. (p. 29)

A psychological model may yield good predictions yet for the purposes of comprehension needs a good underlying story to it. Good predictions serve as a criterion for a model’s accuracy, but comprehension is obtained through a good narrative.

Not every model can be associated with an acceptable narrative, and even if a model is associated with an acceptable narrative, the question remains regarding fitness with reality (e.g., whether people really do make decisions in terms of a gamble). The potential weakness of models may at least partially explain Kahneman’s assertion that “I have always cared more about interesting stories and interesting facts than about precise theories of well-defined and uninteresting domains. Requirements for precision are futile when precision is impossible, and I do not think precision is possible in psychology as we know it today.” This was, as he indicated, the kind of reasoning behind his unpublished piece that applied this critique to prospect theory. A likely corollary is that we should rather use “good” narratives, although the question remains as to what exactly makes a “good” story.

Kahneman’s note raises several issues. First, the emphasis on the values of narrative is to a large extent didactic; that is, a story supposedly assists integrating different aspects of the phenomenon under consideration, thus helping overall comprehension. But in many respects, a mathematical model has similar functions, namely to identify the main variables of a phenomenon and offer a simpler and better comprehension of the phenomenon under investigation. Although an attempt can be made to construct a model as accurately as possible, any thoughtful researcher is aware that a model’s accuracy, certainly in the social sciences, is limited.

Kahneman appropriately noted that stories are much more amenable to laypeople, whereas comprehension of mathematical models is usually restricted to a small group of researchers. However, how stories are understood depends on how they are presented, thus making it difficult to check their validity and reliability. Compared with stories, mathematical models are less vulnerable to framing—the characters used in the model do not have surplus meanings or external connotations.

Exact models cast some restrictions on how they could be tested and interpreted; nonetheless, it can be argued that clarification of the model, or the experimental results conducted to test it, can often be framed and understood in more than one way. Indeed, a major doubt regarding prospect theory emphasized by Kahneman was due to “an intractable difficulty: decisions are influenced by how the choice is presented, and format matters more and more as the complexity of the options increases.”

Format and framing may affect both the comprehension of a narrative as well as the experimental results inferred from a model. Kahneman’s concerns can be generalized to experiments in decision-making, reasoning, or social psychology, all of which are unequivocally susceptible to framing. A conclusion one may derive from our correspondence is that researchers have undermined the importance of framing in the process of experimentally testing models and constructing and explaining narratives.

## Summary

The current article does not offer any clear-cut conclusions. Rather, it tries to point out some essential complications and shortages of psychological research that have often been neglected. Psychological science in the past decades has placed too much emphasis on what may be termed “technical issues,” mainly statistical methods and, more recently, the problem of replication failures (e.g., Simmons et al., 2011; Simons, 2014). Certainly, replication is an obligatory prerequisite of any empirical science. Not undermining the importance of these issues, statistical analyses are often controversial (Cohen, 1990). Similarly, the extent of replication failures and their meaning have also been questioned (e.g., Maxwell et al., 2015). Most imperatively, statistical analyses are necessary but not sufficient—they cannot illuminate the more profound issues of psychological science. The excessive emphasis on statistical issues led, in some respect, to the belittling of other issues, mainly theoretical and conceptual problems.

The correspondence with Kahneman instigated several specific questions about the goals of psychological science: To what extent is it possible to achieve precision in psychological science (and the social sciences in general)? Assuming limited accuracy, what are the best means to obtain psychological knowledge? How should researchers deal with conceptual complications and ambiguities? And given the above limitations, how should we carefully interpret the results of psychological research? The primary purpose of the current article has been to highlight these questions and to remind researchers that, even if there are no definite answers, they should certainly not be overlooked.^4^

A few remaining points should briefly be mentioned. First, as noted by Kahneman, psychologists should remember that precision in psychology is in principle impossible. As Trafimow and Fiedler (2024) pointed out, psychology has always tried to compare itself to physics, specifically what concerns precision. They analyzed a handful of differences between physics and psychology, offering some proposals of what psychology can learn from physics research. Leaving aside that even in today’s physics, specifically quantum physics, perfect precision is impossible, there is a core difference between psychology and physics: As noted earlier, Dawes’s insight that physics theories and models have a corresponding reality whereas psychology does not is crucial. This is probably the major reason why, compared with physics, complete precision in psychology is impossible, and psychology researchers should accept it.

The second point concerns the nature and goals of psychological-science research. Yarkoni and Westfall (2017) claimed that scientific psychology implies “being able both to explain behavior—that is, to accurately describe its causal underpinnings—and to predict behavior—that is, to accurately forecast behaviors that have not yet been observed. In practice, however, these two goals are rarely distinguished” (p. 1100). In terms of the current discussion, models’ main characteristic is their capability to predict, whereas attempts to explain human behavior are facilitated by the use of a narrative. Consistent with Yarkoni and Westfall’s (2017) assertion regarding prediction and the unfolding of causal underpinning associated with a specific behavior, the relationship between models and narratives is intricate.

Both models and stories offer a framework for examining and comprehending new observations and novel hypotheses. Models, if properly constructed, are based on overt assumptions, and any interpretation of the model (or empirical results obtained for testing it) is restricted by the model’s underlying assumptions (and the extent to which they are compatible with reality). Further, regardless of the evidence, one may always contest the assumptions’ veracity. Stories supposedly facilitate comprehension yet can be comprehended in more than one way. There are no unequivocal means for testing their truth, which occasionally can be entirely elusive.

Models and narratives may contribute to enhancing knowledge; each may have its pros and cons. My correspondence with Kahneman suggests that later in his career he tended to believe that the cons of models, at least in psychology, were greater than the pros. It is important to keep in mind that both models and narratives are “mental scaffolds” and not reality or truth itself. Polya (1945) proposed that models and narratives can be viewed as heuristics, or simplification tools, constituting a temporary bridge toward the end goal. Models and stories are both provisional and can be conceived of as temporary mental scaffolds that are used for support until sufficient final comprehension is achieved (which, admittedly, is not well-defined).

One final note: Kahneman concluded, at least later in his career and in our last correspondence, that models offer few benefits for psychological science because of the lack of accurate research—what is important, he maintained, is a comprehensible narrative that reveals and charts interesting observations. I argued that models can be important for the advancement of psychological science; despite their inherent weaknesses and assuming they are properly constructed, models offer a sound framework for systematic research. Kahneman ended his last email to me as follows: “It would seem that each of us believes the other is an intelligent person who, for some incomprehensible reason, is totally misguided. . . . This has been fun. Danny” (D. Kahneman, personal communication).

## Transparency

*Action Editor:* Arturo E. Hernandez

*Editor:* Arturo E. Hernandez

## Notes

1. Kahneman et al. (2021) distinguished between noise that specifically refers to judgment and variability, which is a general characteristic of human behavior. Both noise and variability hinder precision.

2. Quantum physics is exceptional in this regard.

3. Both a paradigm and a model supposedly offer a framework from which the phenomenon (or phenomena) being studied can be viewed. Although a model can be evaluated formally by experimentally testing some predictions derived from it, a paradigm is broader, providing a general structure that cannot be tested directly (except by some instinctive informal judgments).

4. One of the reviewers of this article noted that Kahneman’s view, at least as presented herein, may be conceived as destructive. It would be more accurate to interpret the article as a reminder of the limitations of psychological science, which does not imply that relatively strong explanatory elucidations of psychological phenomena can be developed.
